# The Effect of Time and Storage Environment on the Dimensional Stability of Thermoplastic Resin Post Patterns: An In Vitro Study

**DOI:** 10.1155/ijod/1708729

**Published:** 2026-10-22

**Authors:** Abbasi Benika, Rezaeiyazdi Asal, Mohammadzadeh Mehdi, Ebadian Behnaz, Rezaeikhouzani Negin

**Affiliations:** ^1^ Department of Prosthodontics Faculty of Dentistry Isfahan University of Medical Sciences Isfahan Iran mui.ac.ir; ^2^ Faculty of Dentistry Isfahan University of Medical Sciences Isfahan Iran mui.ac.ir; ^3^ Dental Implants Research Center Department of Prosthodontics Faculty of Dentistry Isfahan University of Medical Sciences Isfahan Iran mui.ac.ir

**Keywords:** dimensional stability, disinfection, post and core, thermoplastic resins

## Abstract

**Background Statement:**

To avoid cross‐contamination, it is essential to disinfect post patterns before fabricating the cast‐metal post. It is of great importance to study the effect of various disinfectants and storage media, as well as time, on the dimensional stability of thermoplastic resin post patterns.

**Methods:**

In this in vitro experimental study, 24 thermoplastic resin patterns were prepared using an extracted single‐rooted mandibular premolar and stored in one of three solutions: water, 0.5% sodium hypochlorite (3

NaOCl), or Ventisept (*n* = 8 per group). Measurements of apical diameter (AD), coronal diameter (CD), and crown–apical length (CAL) were recorded at baseline and after 1, 2, 4, 8, 24, and 48 h using a digital micrometer. A two‐way repeated‐measures analysis of variance (ANOVA) was performed to evaluate the effect of storage medium, time, and their interaction, followed by Bonferroni‐adjusted pairwise comparisons using SPSS version 27. A *p*‐value < 0.05 was considered statistically significant in all tests.

**Results:**

After 48 h, resin patterns stored in each environment significantly expanded compared to their baseline measurements (all *p*  < 0.001) in AD, CD, and CAL dimensions (0.17, 0.25, and 0.06 mm in NaOCl; 0.07, 0.07, and 0.06 mm in water; 0.04, 0.06, and 0.05 mm in Ventisept). Statistically significant differences among storage environments were observed for AD, CD, and CAL. NaOCl showed greater dimensional changes than water and Ventisept for AD and CD (both *p*   < 0.001). Ventisept showed lower dimensional changes than water for AD (*p* = 0.021) and then both water (*p* = 0.025) and NaOCl (*p* = 0.008) for CAL. These expansions occurred rapidly in the first few hours, and the rate of dimensional change decreased at later time points.

**Conclusion:**

Under the conditions tested, storage environment and duration influenced the dimensional stability of thermoplastic resin post patterns. Ventisept was associated with smaller dimensional changes, whereas NaOCl produced greater expansion, particularly in the apical and coronal dimensions. These findings suggest that the choice of storage/disinfection medium and the duration of storage should be considered when thermoplastic resin post patterns are maintained before subsequent laboratory procedures.

## 1. Introduction

Reconstructing endodontically treated teeth is a fundamental treatment plan in modern dentistry. In cases of extensive structural loss, posts are employed to regain retention and support for the core buildup [1, 2]. Among the various post systems, cast‐metal posts are widely used globally due to their reliability and favorable clinical outcomes [3–5]. The resin patterns for cast‐metal posts can be fabricated from a range of materials, including acrylic and thermoplastic resins [6, 7].

Thermoplastic resins are increasingly used to capture the shape of the root canal and fabricate postpatterns because of several advantageous properties. These materials consist of thermoplastic polymer chains that soften when heated and solidify when cooled without undergoing a chemical reaction. Their advantages include being monomer‐free and nonallergenic, having low porosity, good dimensional stability, the ability to be reheated and reused multiple times without chemical alterations, and excellent biocompatibility [8–14]. In addition, thermoplastic resins eliminate polymerization shrinkage associated with conventional autopolymerizing acrylic resins and provide favorable handling characteristics. However, their dimensional stability may be affected by storage conditions and disinfectant solutions before casting, potentially influencing the accuracy of the final restoration. These characteristics have made thermoplastic resins attractive alternatives for the fabrication of post and core patterns [15].

To ensure the health and safety of laboratory technicians, it is necessary to disinfect the contaminated post patterns before fabricating the cast‐metal post. Although the use of disinfecting agents could potentially affect the dimensional stability and marginal precision of the final restoration, it is still an inevitable step in the fabrication process [16, 17].

Several studies have investigated the dimensional stability of acrylic resin patterns under various storage and disinfection conditions. They have found that both the environment and duration of storage could significantly affect the precision of the resin pattern [16, 18]. In Mahshid et al.’s study [17], it was concluded that storing the samples in glutaraldehyde induced contraction, while sodium hypochlorite led to expansion of the resin. Their study also emphasized that dimensional changes over time did not follow a linear trend, complicating predictions of ideal casting time. Fallah‐Tafti et al. [18] indicated that, while all groups experienced minor dimensional changes, water was the most stable environment for storing acrylic resins compared with Deconex and Unident disinfectants. Sabouhi et al. [16] confirmed these findings by investigating dimensional changes over 48 h and observed minimal alteration in water‐stored samples, while significant expansion was observed in those stored in NaOCl. Despite various methodologies, all studies emphasized the importance of choosing appropriate storage and disinfection media to preserve dimensional accuracy. However, limited evidence is available regarding the effects of storage environment and duration on the dimensional stability of thermoplastic resin post patterns.

In this study, the effects of different disinfectants/storage media and storage time on the dimensional stability of thermoplastic resin patterns were evaluated due to the gap in research regarding this topic. The first null hypothesis was that storage medium would have no significant effect on the dimensional stability of thermoplastic resin patterns. The second null hypothesis was that storage time would have no significant effect on the dimensional stability of thermoplastic resin patterns.

## 2. Materials and Methods

### 2.1. Study Design and Tooth Preparation

This experimental in vitro study was conducted in 2024 at the Isfahan University of Medical Sciences. A single‐rooted mandibular premolar, extracted for orthodontic purposes, was selected and stored in a Chloramine solution (0.5%, Merck, Darmstadt, Germany) under refrigeration for 2 days to ensure proper disinfection. The tooth had at least 2 mm of sound coronal structure above the cementoenamel junction (CEJ) and was free of caries, restorations, fractures, or significant wear within this area. Before the experiment, any remaining calculus and debris were removed using a hand sickle scaler (1810H, Denapuya, Tehran, Iran) by engaging the cutting edge of the instrument beneath the apical or lateral edge of the calculus deposit, followed by a controlled vertical pull stroke directed coronally to dislodge the calculus. Then, the tooth was thoroughly cleaned with Prophylaxis Paste (Golchai, Karaj, Iran) and a polish brush (TPC, Melorin Co., China) attached to a low‐speed handpiece (Coxo, Foshan, China). Then the tooth was mounted in autopolymerizing acrylic resin (Akropars, Tehran, Iran) up to the level of the CEJ to provide a stable and standardized position during the subsequent preparation procedures. Straight‐line access to the root canal was achieved using high‐speed fissure burs (Teeskavan, Tehran, Iran). The canal was prepared using a single‐length technique (S1, S2, F1, F2, and F3) with rotary instruments (E‐connect, Eighteeth, Changzhou, China), maintaining a working length of 19 mm from the buccal cusp tip. During canal preparation, irrigation was performed with a 0.9% sodium chloride solution using a 30‐gauge side‐vented irrigation needle. Following this, the canal was obturated using the lateral condensation technique with gutta‐percha (Meta‐Biomed, Cheongju, South Korea) and an eugenol‐free sealer (Master‐dent, Monroe, NC, USA), reaching a working length of 18.5 mm, which was 0.5 mm short of the radiographic apex. The crown was then horizontally sectioned 2 mm above the CEJ using a double‐sided diamond disk (Teeskavan, Tehran, Iran) and separated from the root. Following the working length, gutta‐percha was removed using Peso reamer sizes 1, 2, and 3 (Dentsply Maillefer, Ballaigues, Switzerland) up to 11 mm, leaving 4 mm of material to ensure an apical seal. A periapical radiograph was then obtained to evaluate the canal and verify the quality of the apical seal.

### 2.2. Sample Fabrication

Before fabricating the resin patterns, a lubricant (Vaseline, Unilever, London, UK/Rotterdam, Netherlands) was applied to the canal using a size 30 paper point to facilitate the formation of the thermoplastic resin pattern. The thermoplastic resin post patterns were fabricated using a modified technique based on the method described by Abdul‐Hameed [15]. A commercially available tubular thermoplastic c glue was used for fabrication of the post patterns. The material was softened using a WISEUP 110‐W adjustable‐temperature hot‐melt glue gun (Yiwu Wiseup Hardware Tools Co., Ltd., Yiwu, Zhejiang, China). The glue gun was set at 120 °C, which was selected to provide sufficient softening and flowability of the thermoplastic material to facilitate its adaptation to the prepared root canal space. A small amount of heated resin was placed at the end of a plastic sprue (Pinjet, Angelus, Londrina, Brazil), extending over approximately two‐thirds of the intended post length. The pinjet was then positioned within the prepared post space, left for 5–10 s, and subsequently lifted and repositioned. Subsequently, each resin pattern was carefully examined, and any undercuts were carefully removed using a Morris No. 15 surgical blade (Shanghai, China; manufactured under a German license). Overall, 24 resin samples were fabricated.

### 2.3. Experimental Method

Three dimensions of the specimens, including coronal diameter (CD), apical diameter (AD), and crown–apical length (CAL), were measured using a digital outside micrometer (INSIZE, Model 3109‐25A, Suzhou, China), with a resolution of 1 μm (0.001 mm) and an accuracy of ±0.002 mm. CD was defined as the mesiodistal distance 1.5 mm apical to the coronal margin, AD as the mesiodistal distance 1.5 mm above the apical reference point, and CAL as the distance between the AD reference mark and a second reference mark positioned exactly 8.5 mm coronally. The same instrument and measurement procedure were used at all measurement time points. Measurements were initially recorded in micrometers and subsequently converted to millimeters for statistical analysis and presentation. The observed dimensional changes in the present study (~0.04–0.25 mm at 48 h) were substantially greater than the nominal resolution and stated accuracy of the instrument. The sample size was determined using the formula reported by Sabouhi et al. [16], with a two‐sided significance level of 0.05 (*α* = 0.05), a statistical power of 80% (*β* = 0.20), and an expected difference of 1.5 standard deviations (*δ* = 1.5σ). The calculation yielded a minimum required sample size of 6.98 specimens per group. This value was rounded up to eight specimens per group, resulting in a total of 24 thermoplastic resin post patterns, which were equally allocated among the three storage environments (*n* = 8 per group), including tap water, 0.5% NaOCl (Golrang, Pakshoo Co., Tehran, Iran), or Ventisept Liquid AF (Novin Pak Shargh (NIPCO), Tehran, Iran), an alcohol‐based disinfectant containing ethanol and didecyldimethylammonium chloride (DDAC), a quaternary ammonium compound with broad‐spectrum antimicrobial activity, at 25 ˚C temperature. Measurements of CAL, CD, and AD were repeated at consecutive intervals of 0, 1, 2, 4, 8, 24, and 48 h using the same digital micrometer. Data were recorded in micrometers and subsequently converted to millimeters for analysis and presentation. Measurement reference points (AD, CD, and CAL) were marked on the thermoplastic resin patterns using a permanent marker to ensure consistency in subsequent assessments. Representative images illustrating the experimental procedure, including the prepared tooth and root canal, fabricated thermoplastic resin post pattern, storage environments, and dimensional measurement procedure, are presented in Figure 1.

**Figure 1:**
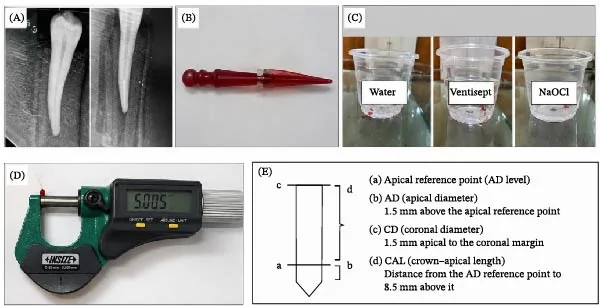
Representative images illustrating the experimental procedure. (A) Radiographic evaluation of the prepared tooth and root canal; (B) fabricated thermoplastic resin post pattern; (C) storage environments (water, Ventisept, and NaOCl); (D) measurement of the resin pattern using a digital micrometer; and (E) schematic representation of the measurement reference points.

### 2.4. Statistical Analysis

At first, the normality of the data distribution was assessed using the Shapiro–Wilk test. A two‐way repeated‐measures analysis of variance (ANOVA) was performed to evaluate the effects of storage medium, time, and their interaction on AD, CD, and CAL. Mauchly’s test of sphericity was used to assess the sphericity assumption, and the Greenhouse–Geisser correction was applied when the assumption of sphericity was violated. Significant effects were further examined using Bonferroni‐adjusted pairwise comparisons, which were selected because they provide control of the family‐wise error rate when multiple pairwise comparisons are performed. Before inclusion in the study, the thermoplastic resin patterns were assessed for predefined exclusion criteria, including visible undercuts, inadequate support of the Pinjet, or apparent dimensional discrepancies in the apical or coronal regions. Exclusion was based on predefined visual criteria and was not determined by statistical testing. None of the 24 fabricated resin patterns met the exclusion criteria; therefore, no samples were excluded from the study.

All the analyses were calculated using Statistical Package for Social Sciences (SPSS) version 27 (Inc., Chicago, IL, USA). A *p*‐value  < 0.05 was considered statistically significant in all tests.

## 3. Results

The Shapiro–Wilk test indicated no significant deviation from normality for AD, CD, or CAL at any measurement time point (all *p*  > 0.05). The means of all measured parameters (AD, CD, and CAL) across the three storage environments are presented in Table 1. Overall mean measurements (mean ± SD) of AD, CD, and CAL across all time points according to the storage environment are summarized in Table 2. The two‐way repeated‐measures ANOVA demonstrated significant main effects of storage medium and time, as well as a significant storage medium × time interaction, for AD, CD, and CAL (all *p*  < 0.001; Table 3). Intergroup comparisons showed significantly higher overall AD and CD measurements in the NaOCl group than in the water and Ventisept groups (*p*  < 0.001). For CAL, a statistically significant difference was also observed among the groups (*p* = 0.017), with the lowest values recorded in the Ventisept group. Pairwise comparisons with Bonferroni adjustment were subsequently performed to identify specific time points that differed significantly within each storage medium (Table 4). Pairwise comparisons of estimated marginal means among the storage environments are presented in Table 5.

**Table 1:** Dimensional changes (mm) of thermoplastic resin patterns stored in water, NaOCl, and Ventisept at various time intervals.

Storage medium	Time (h)	AD (mean ± SD)	CD (mean ± SD)	CAL (mean ± SD)
Water	0	1.01 ± 0.01	1.61 ± 0.03	8.50 ± 0.00
	1	1.02 ± 0.01	1.63 ± 0.03	8.52 ± 0.01
	2	1.03 ± 0.01	1.64 ± 0.03	8.54 ± 0.01
	4	1.05 ± 0.01	1.65 ± 0.03	8.55 ± 0.01
	8	1.07 ± 0.01	1.67 ± 0.03	8.55 ± 0.01
	24	1.07 ± 0.01	1.68 ± 0.03	8.56 ± 0.01
	48	1.08 ± 0.01	1.68 ± 0.03	8.56 ± 0.01

Ventisept	0	1.01 ± 0.01	1.63 ± 0.05	8.50 ± 0.00
	1	1.02 ± 0.01	1.65 ± 0.05	8.51 ± 0.01
	2	1.03 ± 0.01	1.66 ± 0.05	8.52 ± 0.01
	4	1.05 ± 0.01	1.68 ± 0.05	8.53 ± 0.01
	8	1.05 ± 0.01	1.69 ± 0.05	8.54 ± 0.01
	24	1.05 ± 0.01	1.68 ± 0.05	8.55 ± 0.01
	48	1.05 ± 0.02	1.69 ± 0.05	8.55 ± 0.03

NaOCl	0	1.01 ± 0.01	1.62 ± 0.03	8.50 ± 0.00
	1	1.04 ± 0.01	1.72 ± 0.02	8.52 ± 0.01
	2	1.10 ± 0.01	1.78 ± 0.02	8.54 ± 0.01
	4	1.16 ± 0.01	1.83 ± 0.02	8.55 ± 0.01
	8	1.18 ± 0.01	1.85 ± 0.02	8.55 ± 0.01
	24	1.18 ± 0.01	1.86 ± 0.02	8.56 ± 0.01
	48	1.18 ± 0.01	1.87 ± 0.03	8.56 ± 0.01

Abbreviations: AD, apical diameter; CAL, crown–apical length; CD, coronal diameter; SD, standard deviation.

**Table 2:** Overall mean ± SD of AD, CD and CAL measurements (mm) according to the storage environment.

Measurement parameter	NaOCl	Water	Ventisept	*p*‐Value
AD	1.123 ± 0.003^a^	1.048 ± 0.003^b^	1.036 ± 0.003^b^	<0.001
CD	1.789 ± 0.013^a^	1.652 ± 0.013^b^	1.668 ± 0.013^b^	<0.001
CAL	8.540 ± 0.002^a^	8.538 ± 0.002^a^	8.530 ± 0.002^b^	0.017

*Note*: Different superscript letters within each row indicate statistically significant differences between storage environments (*p* < 0.05).

Abbreviations: AD, apical diameter; CAL, crown–apical length; CD, coronal diameter; SD, standard deviation.

**Table 3:** *p*‐Values for overall comparisons of dimensional measurements among storage environments.

Measurement parameter	Storage medium, *p*‐value	Time, *p*‐value	Storage medium × time, *p*‐value
AD	<0.001	<0.001	0.002
CD	<0.001	<0.001	<0.001
CAL	0.017	<0.001	<0.001

*Note:* Greenhouse–Geisser correction was applied when the sphericity assumption was violated. *p*‐value  < 0.05 was considered statistically significant.

**Table 4:** Bonferroni‐adjusted pairwise comparisons of dimensional measurements between baseline and subsequent time points within each storage environment.

Measurement parameter	Storage medium	0 vs. 1 h	0 vs. 2 h	0 vs. 4 h	0 vs. 8 h	0 vs. 24 h	0 vs. 48 h
AD	Water	<0.001	<0.001	<0.001	<0.001	<0.001	<0.001
	Ventisept	<0.001	<0.001	<0.001	<0.001	<0.001	<0.001
	NaOCl	<0.001	<0.001	<0.001	<0.001	<0.001	<0.001

CD	Water	<0.001	<0.001	<0.001	<0.001	<0.001	<0.001
	Ventisept	<0.001	<0.001	<0.001	<0.001	<0.001	<0.001
	NaOCl	<0.001	<0.001	<0.001	<0.001	<0.001	<0.001

CAL	Water	<0.001	<0.001	<0.001	<0.001	<0.001	<0.001
	Ventisept	**0.006**	<0.001	<0.001	<0.001	<0.001	<0.001
	NaOCl	<0.001	<0.001	<0.001	<0.001	<0.001	<0.001

*Note: p*‐Value was adjusted using the Bonferroni correction for six pairwise comparisons within each storage environment. Bold value (*p* = 0.006) indicates statistical significance (*p* < 0.05).

**Table 5:** Pairwise comparisons of estimated marginal means among storage environments.

Measurement parameter	Comparison	Mean difference (mm)	*p*‐Value
AD	Water vs. Ventisept	0.012	0.021
	Water vs. NaOCl	−0.075	<0.001
	Ventisept vs. NaOCl	−0.087	<0.001

CD	Water vs. Ventisept	−0.016	0.377
	Water vs. NaOCl	−0.137	<0.001
	Ventisept vs. NaOCl	−0.121	<0.001

CAL	Water vs. Ventisept	0.008	0.025
	Water vs. NaOCl	−0.002	0.592
	Ventisept vs. NaOCl	−0.010	0.008

*Note:* Pairwise comparisons were based on estimated marginal means using Bonferroni adjustment. *p*‐Values < 0.05 were considered statistically significant.

For AD, the Ventisept group showed significantly lower dimensional changes than the water group (*p* = 0.021), whereas the NaOCl group showed significantly greater dimensional changes than both the water and Ventisept groups (both *p*  < 0.001). For CD, the NaOCl group showed significantly greater dimensional changes than both the water and Ventisept groups (both *p*  < 0.001), whereas no significant difference was observed between the water and Ventisept groups (*p* = 0.377). For CAL, the Ventisept group showed significantly lower dimensional changes than both the water (*p* = 0.025) and NaOCl (*p* = 0.008) groups, whereas no significant difference was observed between the water and NaOCl groups (*p* = 0.592).

For AD, the patterns stored in water, Ventisept, and NaOCl showed expansions of 0.01, 0.01, and 0.03 mm, respectively, within 1 h (all *p*  < 0.001). The expansion continued significantly up to 8 h, reaching 0.06, 0.04, and 0.17 mm compared with baseline, respectively (all *p*  < 0.001). Thereafter, the rate of dimensional change decreased at later time points. After 48 h, AD had increased by 0.07, 0.04, and 0.17 mm in the water, Ventisept, and NaOCl groups, respectively, compared with baseline (all *p*  < 0.001). For CD, the patterns stored in water, Ventisept, and NaOCl showed expansions of 0.02, 0.02, and 0.10 mm, respectively, within 1 h (all *p*  < 0.001). The expansion remained significant up to 4 h, reaching 0.04, 0.05, and 0.21 mm compared with baseline, respectively (all *p*  < 0.001). Thereafter, the rate of dimensional change decreased at later time points. After 48 h, CD had increased by 0.07, 0.06, and 0.25 mm in the water, Ventisept, and NaOCl groups, respectively (all *p*  < 0.001). For CAL, the patterns stored in water, Ventisept, and NaOCl showed expansions of 0.02, 0.01, and 0.02 mm, respectively, within 1 h (*p*  < 0.001 for water and NaOCl; *p* = 0.006 for Ventisept). Thereafter, CAL continued to increase, although the rate of change decreased at later time points. After 48 h, CAL had increased by 0.06, 0.05, and 0.06 mm in the water, Ventisept, and NaOCl groups, respectively, compared with baseline (all *p*  < 0.001).

## 4. Discussion

This experimental study evaluated the effects of storage medium (NaOCl, water, and Ventisept) and storage time on the dimensional stability of thermoplastic resin post patterns. The results revealed statistically significant dimensional changes in AD, CD, and CAL over time in all three storage environments, rejecting the null hypotheses. A comparison of different storage media showed that NaOCl produced the greatest dimensional changes in AD and CD. For AD, the Ventisept group showed significantly lower dimensional changes than the water group. For CD, no significant difference was observed between the water and Ventisept groups. For CAL, the Ventisept group showed significantly lower dimensional changes than both the water and NaOCl groups, whereas no significant difference was observed between the water and NaOCl groups. These findings indicate that both the storage environment and duration of exposure may influence the dimensional stability of thermoplastic resin patterns.

Regarding the effect of storage medium, the greatest dimensional changes were generally observed in the NaOCl group. For AD and CD, the overall dimensional changes were significantly greater in NaOCl than in water and Ventisept. For CAL, the Ventisept group demonstrated significantly lower dimensional values than both water and NaOCl, whereas no significant differences were observed between water and NaOCl groups.

The effect of storage time was also evident across all three dimensions. The greatest changes generally occurred during the early stages of storage, followed by a gradual reduction in the rate of dimensional change. For AD, significant expansion was observed up to 8 h, whereas for CD, significant expansion continued up to 4 h. In CAL, the expansion increased over time before showing a lower rate of change at later intervals. Overall, the rate of dimensional change decreased at later time points, although some further dimensional changes were still observed up to 48 h. The observed dimensional expansion of thermoplastic resin patterns may be related to the interaction between the thermoplastic resin and the storage environment over time, including possible moisture uptake [16].

The pattern of dimensional change also differed according to the storage medium, indicating that the effect of time was not independent of the storage environment. The NaOCl group exhibited the greatest magnitude of change for AD and CD. Ventisept showed significantly lower dimensional changes than water for AD and significantly lower changes than both water and NaOCl for CAL, whereas no significant difference was observed between water and Ventisept for CD.

This difference in the temporal pattern of dimensional change may reflect differences in the chemical composition and interaction of each storage medium with the resin material. Therefore, interpretation of dimensional stability should consider both the duration of storage and the specific environment in which the resin pattern was maintained.

The lower dimensional change observed in the Ventisept group compared with water may be attributable, at least in part, to the presence of ethanol in its formulation [16]; however, this proposed mechanism was not directly evaluated in the present study. In contrast, the greater dimensional changes observed in the NaOCl group may be related to chemical interactions between the oxidizing solution and the polymer matrix, potentially affecting the polymer structure and moisture‐related dimensional behavior. However, the underlying mechanism was not directly investigated in the present study. These changes may contribute to dimensional expansion and deformation during storage, especially with prolonged exposure times [19]. DDAC exerts its antimicrobial action primarily through the disruption of microbial cell membranes rather than through strong oxidative reactions [20]. Therefore, compared with sodium hypochlorite, DDAC‐containing disinfectants may exhibit lower chemical interaction with the thermoplastic resin matrix, which could contribute to the lower dimensional changes observed in the Ventisept group. The findings of the present study are consistent with previous reports indicating that sodium hypochlorite can adversely affect the properties of dental materials [21]. The findings of the present study can be compared with previous investigations of the dimensional stability of resin patterns, although differences in resin composition and experimental conditions should be considered. Sabouhi et al. [16] investigated acrylic resin patterns stored in air, water, and 5% NaOCl and reported rapid dimensional changes during the first hour, followed by slower changes at subsequent time points. This temporal pattern was generally consistent with the present findings. However, the acrylic resin patterns stored in water and air in their study showed contraction, whereas expansion was observed in the present study, which may be attributable to the differences in the resin materials used. Their study also demonstrated greater dimensional changes in the NaOCl group, which is consistent with the present findings.

Mahshid et al. [17] evaluated the effects of NaOCl and glutaraldehyde under dry and humid storage conditions using Duralay acrylic resin patterns. Expansion was observed in the NaOCl group, which is consistent with the present findings. However, their study reported contraction in samples stored in glutaraldehyde and did not identify significant differences between the moist environments. These findings differ from the present results, in which differences among storage media were observed for some dimensional measurements. Differences in resin composition, storage media, and measurement intervals may contribute to these discrepancies. In addition, Mahshid et al. [17] reported that dimensional changes did not follow a consistent pattern over time, whereas the present study demonstrated a general trend toward a lower rate of dimensional change at later time points. Fallahtafti et al. [18] evaluated acrylic resin patterns stored in water and disinfecting solutions (Deconex and Unident) and reported that water produced the smallest dimensional changes in Duralay patterns. This finding was generally consistent with the present study, in which water showed relatively limited dimensional changes compared with those of NaOCl, particularly for AD and CD. Nevertheless, direct comparison should be made cautiously because the resin material and disinfectant solutions differed between the two studies.

Taken together, the present findings suggest that the dimensional stability of thermoplastic resin post patterns is influenced by both the storage environment and the duration of storage. NaOCl resulted in the greatest dimensional changes, whereas Ventisept generally produced the smallest changes. The changes were more pronounced during the earlier storage intervals, and the rate of dimensional change decreased at later time points. These findings highlight the importance of considering both the type of disinfectant or storage medium and the duration of exposure when thermoplastic resin patterns are stored before subsequent laboratory procedures.

## 5. Limitation

This study has several limitations, including its in vitro design and limited sample size. Further studies with larger sample sizes and clinical or laboratory‐based designs are needed to confirm these findings and evaluate their clinical relevance, including post adaptation and retention after casting and cementation. In addition, the use of a contact digital micrometer may have introduced a small degree of measurement‐related error, as contact force was not independently quantified. Future studies may consider validated noncontact optical methods for dimensional assessment.

## 6. Conclusion

This in vitro study demonstrated that the dimensional stability of thermoplastic resin post patterns was significantly influenced by storage environment and storage time. Among the storage media evaluated, NaOCl produced the greatest dimensional changes in AD and CD, whereas Ventisept generally exhibited the smallest dimensional changes. Dimensional changes were more pronounced during the early storage intervals, with a reduced rate of change at later time points. These findings suggest that both the storage medium and duration of exposure should be considered when thermoplastic resin post patterns are maintained before subsequent laboratory procedures. Future studies with larger sample sizes and evaluation through subsequent casting and cementation procedures are warranted to further clarify the clinical implications of storage conditions on post adaptation and restoration longevity.

## Funding

This study was supported by the Isfahan University of Medical Sciences, 3403170.

## Ethics Statement

The study protocol was approved by the Ethics Committee of Isfahan University of Medical Sciences (Approval Number IR.MUI.DHMT.REC.1403.066).

## Consent

Informed consent was not applicable as only extracted teeth were used in an in vitro setting.

## Conflicts of Interest

The authors declare no conflicts of interest.

## Data Availability

The data supporting the findings of this study are available from the corresponding author upon reasonable request.
